# Quasi-random square gabor spiral zone plates for high-order diffraction suppression and multi-functional optical vortex generation

**DOI:** 10.1371/journal.pone.0340251

**Published:** 2026-02-05

**Authors:** Huakui Hu, Jiangtao Ding, Weifeng Wu, Huajie Xu, Guoping Shi, Hailiang Li

**Affiliations:** 1 Research Center of Integrated Circuits Design and semiconductor processing material, Chizhou College, Chizhou, China; 2 Anhui Research Center of Semiconductor Industry Generic Technology, Chizhou, China; 3 College of Mechanical and Electrical Engineering, Chizhou College, Chizhou, China; 4 Key Laboratory of Microelectronic Devices and Integrated Technology, Institute of Microelectronics, Chinese Academy of Sciences, Beijing, China; Purdue University, UNITED STATES OF AMERICA

## Abstract

Due to carrying the orbital angular momentum, Optical vortices generated by spiral zone plates, have become an important tool for studying physics and detecting matter. However, spiral zone plates, limited by its inherent structure, struggle to integrate multiple functionalities including efficient high-order diffraction suppression, flexible generation of complex vortices, and compatibility with standard planar fabrication processes into a single element to meet current composite demands. By converting the three-dimensional structure of a Gabor spiral zone plates into a two-dimensional structure, we propose a single-focus spiral zone plates with an approximately sinusoidal transmittance, termed the quasi-random square Gabor spiral zone plates (QSGSZPs). Both theoretical analysis and experimental results demonstrate that the QSGSZPs not only effectively achieve single-order diffraction but also enable the generation of complex vortex structures, including flower-shaped optical vortex lattices and vortex twins, through topological charge modulation. The focusing properties of the QSGSZPs with different parameters were also investigated, and its potential applications in edge-enhanced imaging and optical communications were demonstrated. This element with its unique properties is expected to find widespread applications in a variety of fields.

## 1. Introduction

Vortex [1–5] is ubiquitous in nature, observable in contexts ranging from the grand spiral structures of galaxies and the powerful gravitational fields of black holes to the complex dynamic patterns of oceanic circulation. These phenomena are typically characterized by a central singularity [6,7], a point at which physical quantities become undefined, leading to unique local behaviors and energy distribution patterns. Singularities exist not only on macroscopic scales but also manifest in the microscopic world. The light beams that possess the singularity is known as optical vortex or vortex beams [8–10]. The optical vortex exhibits a helical phase structure and a phase singularity along the optical axis, resulting in a ring-shaped intensity distribution with zero central intensity. The phase structure of an optical vortex can be mathematically characterized by the function exp(*ilφ*), where *l* represents the topological charge and *φ* denotes the azimuthal angle. Owing to its unique phase singularity, optical vortex exhibits considerable application potential and distinctive advantages across various fields, including optical communications [11,12], quantum information [13], microscopy [14], and particle manipulation [15], among others. In recent years, a variety of methods have been developed to generate optical vortex [16–25], such as spiral phase plates [16], computer-generated holograms [17], q-plates [18], metasurfaces [19], parametric equations [20–22] and diffractive optical elements [23]. Among these, the spiral zone plates (SZPs) [26,27], as a type of diffractive optical element, has gained significant interest owing to its compact configuration and high diffraction efficiency. In particular, the SZPs can be widely utilized in the X-ray regions, where many other components struggle to operate effectively.

However, the SZPs exhibits a planar periodic structure analogous to that of the Fresnel zone plates (FZPs), which inherently induces high-order diffraction [28]. Consequently, the SZPs generates optical vortices not only at the position of focal length *f*, but also at its odd fractional distances (e.g., *f*/3, *f*/5, *f*/7). In most practical applications, only the first-order diffraction is needed, and the existence of high-order diffraction introduces unwanted background noise, which may lead to imaging artifacts and degradation of the overall resolution in optical systems. In recent years, single diffractive optical elements have been proposed and realized for the suppression of high-order diffraction [29–32]. Photon sieves, which uses a multitude of pinholes in place of circular rings, are capable of suppressing high-order diffraction by several orders of magnitude [29]. The generalized binary spiral zone plates, which is designed by a trained feedforward neural network, can reduce the intensity of high-order diffraction to as low as 0.2% of the required first-order diffraction [30]. Moreover, The complex vortices has gradually attracted more attention, such as the flower-shaped optical vortex lattices and vortex twins [33,34]. These structures are typically generated through the interference of multiple Laguerre-Gaussian beams, possessing multiple topological characteristics and angular self-reconstruction capabilities, which hold significant value across various application domains. For instance, in optical communications, they enable the construction of high-dimensional encoding spaces, paving new pathways to enhance channel capacity and system security [35]. However, despite their respective strengths in specific performance metrics, existing diffractive optical elements still lack a single component capable of simultaneously meeting all three requirements of efficient high-order diffraction suppression, flexible generation of complex vortices, and compatibility with standard planar fabrication processes.

Here in this paper, a novel spiral zone plates, called quasi-random square Gabor spiral zone plates (QSGSZPs), is designed by transforming the three-dimensional structure of a Gabor spiral zone plates into a two-dimensional planar structure. Both experimental and theoretical results consistently show that the QSGSZPs can achieve single-order diffraction and generate complex vortices by modulating the topological charge, including the flower-shaped optical vortex lattice and vortex twins. In addition, the influences of different parameters on the focusing characteristics of the QSGSZPs are systematically investigated, and its application potential in the fields of edge-enhanced imaging and optical communications is evaluated.

## 2. Design

For the conventional FZPs, its transmittance function can be written as:


$$T_{FZPs}(r)=\left\{ \begin{array}{c} 1\;\;\;\;\;\;\;\;\;\;\;\;\;sin\left(\pi r^{\text{2}}/\lambda f\right)\geq 0\\ 0\;\;\;\;\;\;\;\;\;\;\;\;\;sin\left(\pi r^{\text{2}}/\lambda f\right)\mathrm{\backslash textless}0 \end{array}\right.$$ (1)

**Fig 1:**
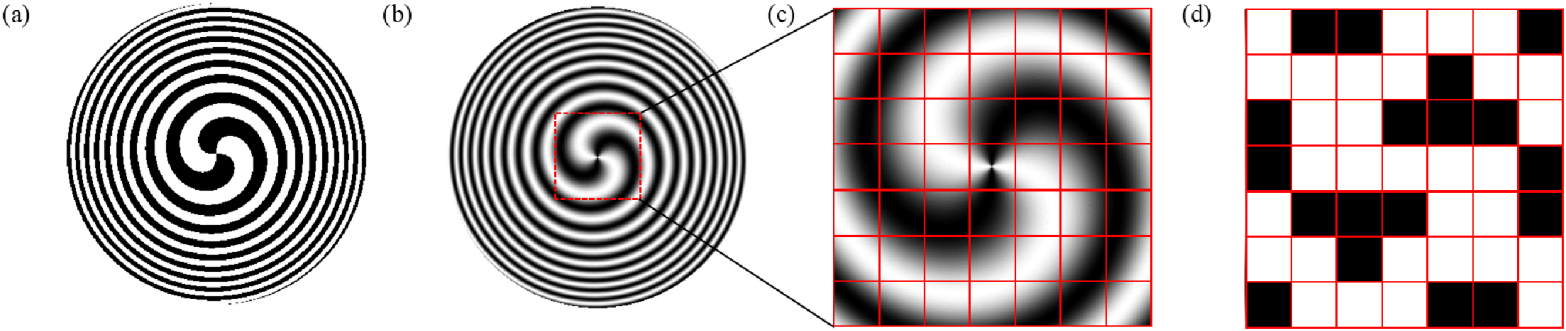
Schematic diagrams of the designed elements and the design process of the QSGSZPs. **(a)** Structure of the SZPs. **(b)** – **(d)** Design workflow for the proposed QSGSZPs: (b) the continuous transmittance profile of the GaborSZPs, (c) the partitioning of the profile into discrete square cells, and (d) the final binary structure of the QSGSZPs after the binarization operation.


Where *λ* is the wavelength and *f* represents the focal length. The SZPs is constructed by introducing a spiral phase structure into a traditional FZPs, as shown in Fig 1(a). Its transmittance function can be expressed as:

$$T_{SZPs}\left(r,\varphi \right)=\left\{ \begin{array}{c} 1\;\;\;\;\;\;\;\;\;\;\;\;\;sin\left(l\varphi -\pi r^{\text{2}}/\lambda f\right)\geq 0\\ 0\;\;\;\;\;\;\;\;\;\;\;\;\;sin\left(l\varphi -\pi r^{\text{2}}/\lambda f\right)\mathrm{\backslash textless}0 \end{array}\right.$$ (2)

Where (*r*, *φ*) is the polar coordinates on the diffraction plane, and *l* represents the topological charge, which defines the order of the phase singularity in an optical vortex and determines the magnitude of the orbital angular momentum (OAM) carried by a photon.

According to the expression of the SZPs, the transmittance function is binary (0 or 1), and its distribution can be approximated as a square wave. According to Fourier analysis, a square wave can be decomposed into a fundamental frequency component (corresponding to the designed first-order focal length *f*) and a series of odd-order higher-order harmonics (such as the third and fifth harmonics, corresponding to focal lengths like *f*/3, *f*/5, etc.). These higher-order harmonics physically manifest as unavoidable high-order diffraction foci. If high-order diffraction can be effectively eliminated and its application process simplified, the SZPs is expected to achieve broader applications in numerous fields. Inspired by the ability of the Gabor zone plates to completely suppress high-order diffraction [36], a novel single-order spiral zone plates, named the Gabor spiral zone plates (GaborSZPs), is proposed by combining its high-order diffraction suppression capability with the radial Hilbert transform function. As shown in Fig 1(b), its transmittance function can be expressed as:


$$T_{GaborSZPs}\left(r,\varphi \right)=\frac{\text{1}}{\text{2}}-\frac{\text{1}}{\text{4}}\left[\exp\left(\frac{i\pi r^{2}}{\lambda f}+il_{1}\varphi \right)+\exp\left(-\frac{i\pi r^{2}}{\lambda f}+il_{2}\varphi \right)\right]$$ (3)

As indicated by Eq. (3), the GaborSZPs possesses a continuous and approximately sinusoidal transmittance function, whose Fourier spectrum contains only a single fundamental frequency component without higher-order harmonics. Consequently, when this structure is illuminated by a plane wave, it generates only the diffraction field corresponding to this fundamental frequency. This fundamental frequency component manifests physically in the form of a pair of conjugate foci, comprising a real focus and a virtual focus positioned on opposite sides of the optical element, which constituting the complete diffraction field generated by the sinusoidal transmittance function. Therefore, the GaborSZPs enables single-focus diffraction without high-order diffraction. However, the transmittance of the GaborSZPs is not binary (0 or 1) but exhibits continuous variation between 0 and 1, indicating a three-dimensional sinusoidal distribution characteristic in its structure. This property makes such components challenging to fabricate using standard planar semiconductor fabricated processes.

To solve this issue, quasi-random square Gabor spiral zone plates (QSGSZPs) is proposed. The specific procedure is illustrated in Figs 1 (b)-1(d). Firstly, the transmittance of the GaborSZPs is calculated based on Eq (3), and the corresponding transmittance pattern is generated. Subsequently, this pattern is divided into a large number of small square cells, as shown in Fig 1(c), where the average transmittance of each cell ranges between 0 and 1. Finally, a binarization process is applied to each cell using Eq (4), resulting in the designed QSGSZPs, whose structure is depicted in Fig 1(d). This discretization design effectively transfers the single-focus diffraction characteristics of ideal GaborSZPs to the manufacturable two-dimensional planar architecture of the QSGSZPs while fully preserving spiral phase modulation capability, thereby achieving practical device manufacturability without sacrificing optical performance.


$$T_{QSGSZPs}\left(i,j\right)=\left\{ \begin{array}{c} 1\;\;\;\;\;\;\;\;\;\;rand\leq T_{GaborSZPs}\left(i,j\right)\\ 0\;\;\;\;\;\;\;\;\;\;rand(T_{GaborSZPs}\left(i,j\right) \end{array}\right.$$ (4)

Where *T*_GaborSZPs_(*i*, *j*) and *T*_QSGSZPs_(*i*, *j*) represent the transmittance of the square cell at the i-th row and j-th column of the GaborSZPs and QSGSZPs, respectively. The rand is used to generate a random number, with its value ranging between 0 and 1.

## 3. Focusing properties of the QSGSZPs

When a plane wave is incident on the QSGSZPs, the complex amplitude distribution of the generated vortex beam on the observation plane can be expressed as:


$$\begin{array}{c} Y_{out}(x_{2},y_{2})=\frac{\exp(jkf)\exp\left[\frac{jk}{2f}({x_{2}}^{2}+{y_{2}}^{2})\right]}{j\lambda f}\\ \times 2DCZT\left\{T_{QSGSZPs}(x_{1},y_{1})\times \exp\left[\frac{j\pi }{\lambda z}({x_{1}}^{2}+{y_{1}}^{2})\right]\right\} \end{array}$$ (5)

Where *z* is the propagation distance, (x_1_, y_1_) and (x_2_, y_2_) are the cartesian coordinates on the QSGSZPs plane and the observation plane, respectively, 2D CZT denotes the two-dimensional chirp z-transform.

**Fig 2:**
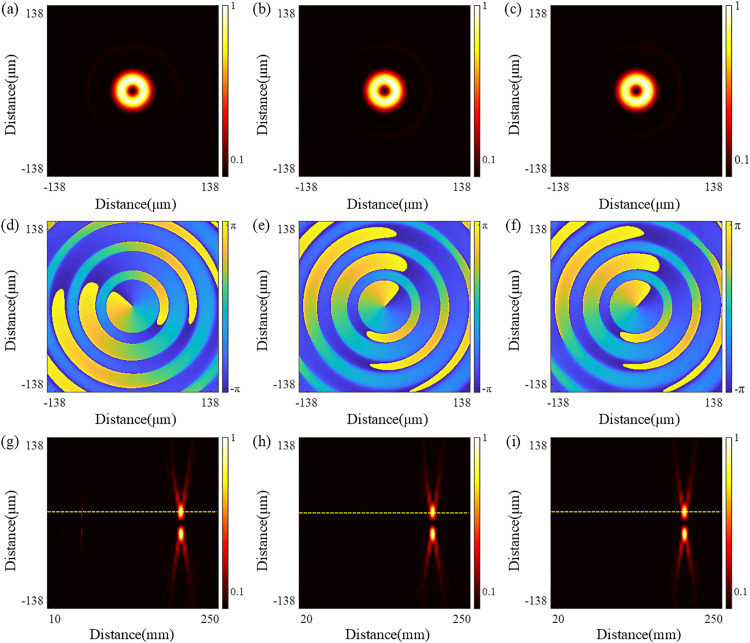
The comparison of the simulated diffraction performance. **(a)** – **(c)** The normalized intensity distributions for the SZPs, GaborSZPs, and QSGSZPs at the focal plane, respectively. The x and y axes represent the spatial coordinates, and the color bar indicates the normalized intensity. **(d)** – **(f)** The corresponding spiral phase distributions corresponding to **(a)** – **(c)**. The color bar indicates the phase value in radians. **(g)** – **(i)** The cross-sectional diffraction intensity distributions along the propagation from *z* = 10 mm to *z* = 250 mm corresponding to **(a)** – **(c)**. The x-axis represents the propagation distance *z*, and the y-axis represents the radial distance from the optical axis.

**Fig 3:**
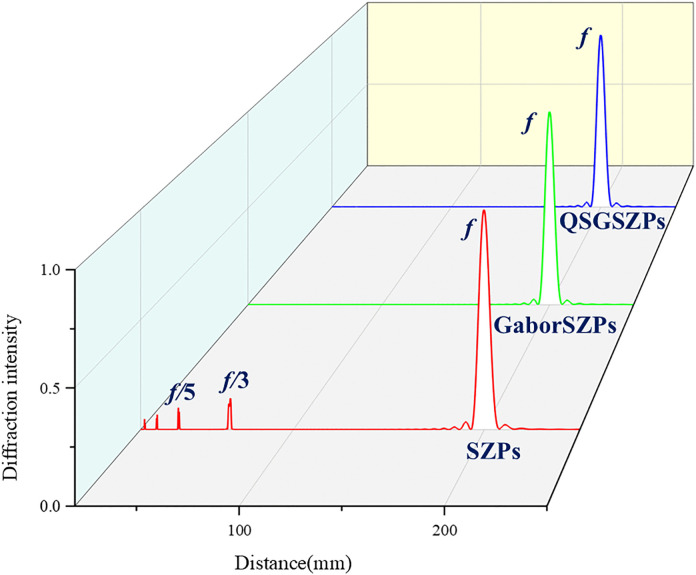
The axial simulated diffraction intensity of the yellow dashed line in Figs 2 (g) –2(i). The x-axis represents the propagation distance *z* and the y-axis represents the normalized diffraction intensity on the optical axis.


Before the simulation, it should be noted that the parameters for the QSGSZPs are set as: *λ* = 632.8 nm, the size *S* of square = 2.5 μm × 2.5 μm, *l*_1_ = 2, *l*_2_ = 2, and *f* = 200 mm. Fig 2 shows the normalized far-field diffraction intensity distribution, phase distribution, and axial diffraction intensity distribution for the SZPs, GaborSZPs and QSGSZPs. As seen in Figs 2 (a) - 2(c), the SZPs, GaborSZPs and QSGSZPs all generate optical vortex with a circularly symmetric dark-core structure at the focal plane. The phase distribution, as illustrated in Figs 2 (d) - 2(f), exhibits the expected spiral structure. For each full revolution around the center along the radial direction, the phase undergoes a 4π jump, resulting in a phase singularity with a topological charge of *l* = 2 at the beam center. It is noteworthy that the phase distributions of the three elements are highly consistent, indicating that the randomly distributed square structure does not cause significant phase distortion. The cross-sectional diffraction intensity of the these elements along the propagation from z = 10 mm to z = 250 mm is shown in Figs 2 (g) - 2(i). The SZPs generates the target optical vortex at the designed focal length *f*, but multiple low-intensity vortices also appear near positions such as *f*/3 and *f*/5, indicating the presence of significant high-order diffraction. In contrast, the GaborSZPs produces the desired optical vortex only at the focal length *f*, with no vortex structures detected at other positions, demonstrating its effective suppression of high-order diffraction. Based on the structural design of GaborSZPs, the QSGSZPs inherits similar diffraction characteristics, preserving only the desired first-order diffraction vortex at the focal length *f* and thereby significantly enhancing diffraction purity. To further quantitatively evaluate the performance of the QSGSZPs, Eq (6) was employed to analyze the diffraction characteristic. The proposed QSGSZPs improves the signal-to-noise ratio (SNR) from 17 dB in the SZPs to 79 dB, corresponding to an increase of approximately 62 dB in Fig 3. These quantitative results collectively confirm that the QSGSZPs significantly suppresses high-order diffraction, validating its effectiveness in reducing background noise.

$$SNR=20\times \log_{10}\left(\frac{I_{1}}{I_{3}}\right)$$ (6)

Where *I*_1_ and *I*_3_ represent the normalized peak intensities at the target first-order and third-order foci, respectively.

**Fig 4:**
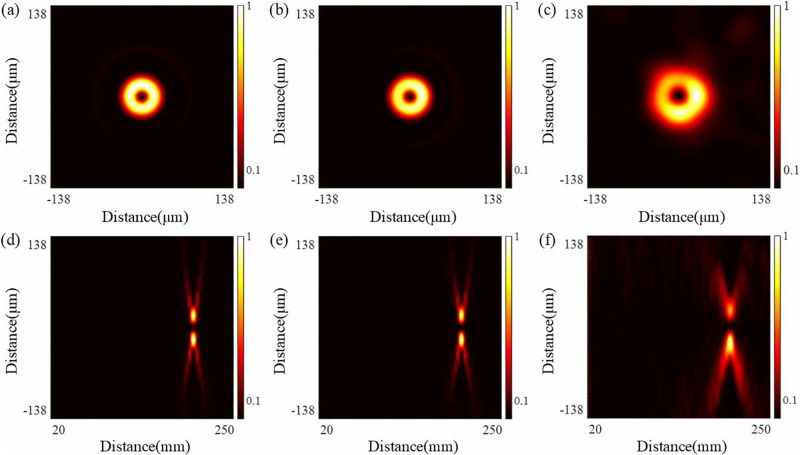
Simulation study on the effect of the square size *S* on the performance of the QSGSZPs. **(a)** – **(c)** Normalized intensity distributions at the focal plane for *S* = 2.5 μm × 2.5 μm, 5 μm × 5 μm and 10 μm × 10 μm, respectively. **(d)** – **(f)** The cross-sectional diffraction intensity of the QSGSZPs along the propagation from *z* = 10 mm to *z* = 250 mm corresponding to **(a)** – **(c)**.

For the QSGSZPs, its structure consists of a large number of randomly arranged squares, and this randomness causes the transmittance function to approximate a sinusoidal distribution. Among these, the size *S* of square is a key parameter affecting the performance of the QSGSZPs. Fig 4 shows that as the size *S* of square increases, the quality of the optical vortex gradually deteriorates, the ability to suppress high-order diffraction weakens, and background noise significantly intensifies. This phenomenon can be attributed to the fact that a larger size *S* of square leads to a greater discrepancy between the fitted transmittance of the QSGSZPs and the transmittance function of an ideal GaborSZPs, making it difficult to effectively suppress high-order diffraction. Therefore, the smaller the subdivision size, the greater the number of squares obtained, and the closer the fitted transmittance approaches the transmittance function of a GaborSZPs. However, it should be noted that excessively small square would significantly increase fabricated difficulty, requiring full consideration of laboratory processing capabilities in practical design.

**Fig 5:**
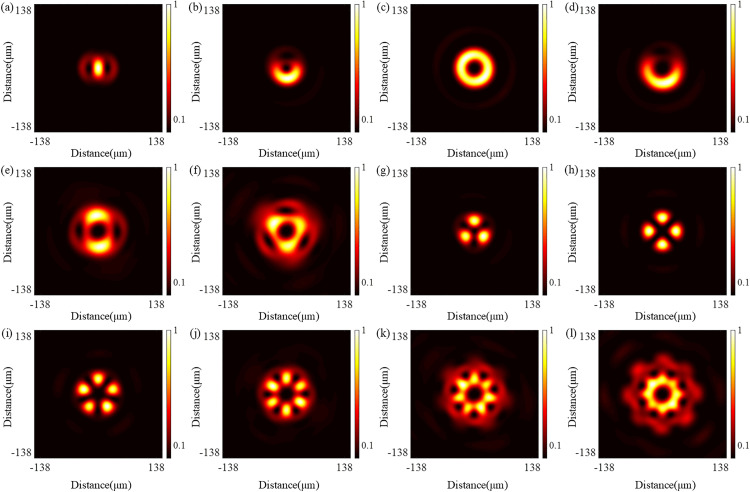
Simulated intensity distributions of complex vortices generated by the QSGSZPs with different topological charges (*l*_1_, *l*_2_). **(a)** – **(l)** The topological charges of the QSGSZPs are as follows: (*l*_1_ = 2, *l*_2_ = 0), (*l*_1_ = 2, *l*_2_ = 1), (*l*_1_ = 2, *l*_2_ = 2), (*l*_1_ = 2, *l*_2_ = 3), (*l*_1_ = 2, *l*_2_ = 4), (*l*_1_ = 2, *l*_2_ = 5), (*l*_1_ = 2, *l*_2_ = −1), (*l*_1_ = 2, *l*_2_ = −2), (*l*_1_ = 2, *l*_2_ = −3), (*l*_1_ = 2, *l*_2_ = −4), (*l*_1_ = 2, *l*_2_ = −5), and (*l*_1_ = 2, *l*_2_ = −6), respectively.The parameters *l*_1_ is fixed at 2 while *l*_2_ is varied.

According to the design principle of the QSGSZPs, the optical vortex generated at the focal plane is jointly determined by the topological charges *l*_1_ and *l*_2_, thus it is essential to systematically investigate the modulation characteristics of optical vortex by the topological charges *l*_1_ and *l*_2_. As clearly shown in Fig 5, by adjusting the combination of key parameters *l*_1_ and *l*_2_, the QSGSZPs can not only generate conventional optical vortex but also demonstrate a unique capability to surprisingly produce complex vortex structures such as the flower-shaped optical vortex lattice and vortex twins. Notably, such complex vortices break the inherent pattern of strictly annular intensity distribution at the center of the traditional optical vortex. Their characteristics, such as non-zero central intensity or incomplete annular intensity distribution, significantly enhance the detection capability of rotating targets. Furthermore, the vortex region gradually expands with increasing topological charges, which is consistent with the scaling behavior observed in traditional optical vortex.

**Fig 6:**
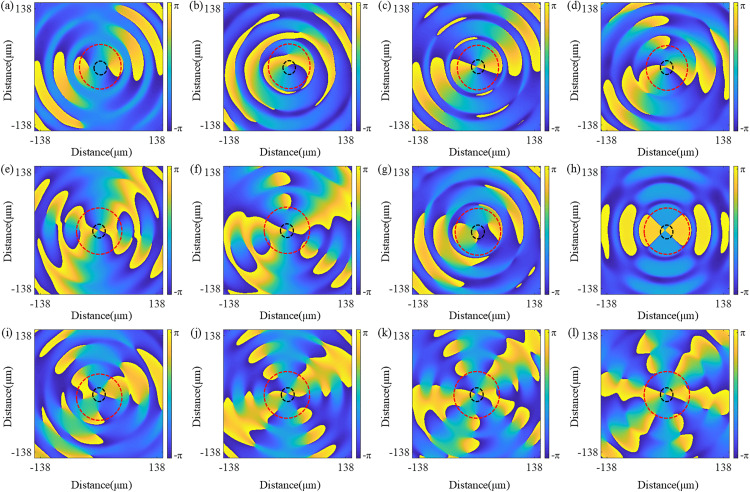
Simulated phase distributions corresponding to the intensity patterns shown in Fig 5. **(a) – (l)** The topological charges of the QSGSZPs are as follows: (*l*_1_ = 2, *l*_2_ = 0), (*l*_1_ = 2, *l*_2_ = 1), (*l*_1_ = 2, *l*_2_ = 2), (*l*_1_ = 2, *l*_2_ = 3), (*l*_1_ = 2, *l*_2_ = 4), (*l*_1_ = 2, *l*_2_ = 5), (*l*_1_ = 2, *l*_2_ = −1), (*l*_1_ = 2, *l*_2_ = −2), (*l*_1_ = 2, *l*_2_ = −3), (*l*_1_ = 2, *l*_2_ = −4), (*l*_1_ = 2, *l*_2_ = −5), and (*l*_1_ = 2, *l*_2_ = −6), respectively. The topological charges accumulated along the black and red circles correspond to the minimum and maximum values of *l*_1_ and *l*_2_, respectively.


The formation mechanism of complex vortex structures stems from the physical nature of their optical fields, namely the coherent superposition of vortex modes corresponding to two different topological charges. Mathematically, the complex amplitude field distribution generated by the QSGSZPs at the focal plane can be expressed by Eq. (7). From a physical mechanism perspective, this superposition leads to dramatic changes in the wavefront. When *l*_1_ ≠ *l*_2_, two wavefronts carrying different OAMs interfere with each other, causing the original on-axis phase singularity to split and generate new and stably arranged off-axis phase singularities. These off-axis singularities correspond to dark cores in the intensity distribution and determine their number, while the regions of maximum interference between them form bright lobe structures, collectively constituting complex patterns such as flower-shaped optical vortex lattice. The spiral superposition can be observed from the phase distribution in Fig 6. Along the closed red dashed circle, a phase of 2π*max(*l*_1_, *l*_2_) is accumulated, while along the closed black dashed circle, a phase of 2π*min(*l*_1_, *l*_2_) is accumulated. This indicates the redistribution of the phase structure through superposition and confirms the coexistence of multiple topological charges. Particularly noteworthy is that the number of bright peaks in the amplitude distribution equals |*l*_1_ - *l*_2_|, which further demonstrates the coexistence of multiple topological charges. Therefore, through systematic adjustment of *l*_1_ and *l*_2_, one can not only precisely control the number and spatial arrangement of phase singularities, but also achieve control over the intensity distribution through amplitude modulation, ultimately enabling precise customization of optical fields ranging from conventional vortex to various complex structures.

$$U\left(r,\varphi \right)\propto \left[\exp\left(il_{1}\varphi \right)+\exp\left(il_{2}\varphi \right)\right]\times A\left(r\right)$$ (7)

where *A*(r) represents the radial envelope function.

**Fig 7:**
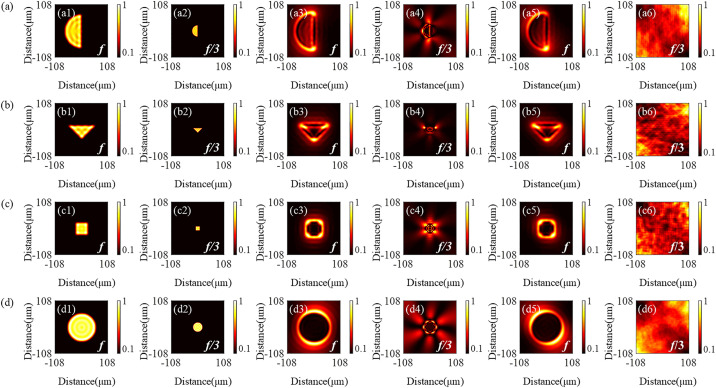
Simulated edge-enhanced imaging performance. A comparative analysis of the imaging produced by the FZPs (Figs 7 (a1) – (d1) and (a2) – (d2)), SZPs (Figs 7 (a3) – (d3) and (a4) – (d4)), and QSGSZPs (Figs 7 (a5) – (d5) and (a6) – (d6)) under various aperture shapes. For each aperture shape (semicircular, triangular, rectangular, circular), the images show the intensity distribution at the position of first-order (*f*) and third-order (*f*/3) focal planes.

Edge enhancement imaging [37] is one of the important characteristics of optical vortex, which can significantly improve the imaging contrast at the boundaries of target objects and effectively overcome the limitations of traditional imaging techniques, such as edge blurring. However, the presence of high-order diffraction for the SZPs introduces additional artifacts during the imaging, thereby reducing the contrast sensitivity. To validate the imaging performance of the proposed QSGSZPs, numerical simulations were conducted on small apertures with different geometries (semicircular, circular, rectangular, triangular). As shown in Fig 7, at the focal plane, both the SZPs and the QSGSZPs clearly reveal the boundary contours of these apertures, and their edge enhancement effects are significantly superior to those of the FZPs. It is noteworthy that both the FZPs and the SZPs exhibit noticeable artifacts at the position of *f*/3, whereas only background noise is observed at the same position for the QSGSZPs, with its artifacts effectively suppressed. Therefore, the excellent imaging purity demonstrated by the QSGSZPs provides a potential technical pathway for achieving high-resolution imaging.

**Fig 8:**
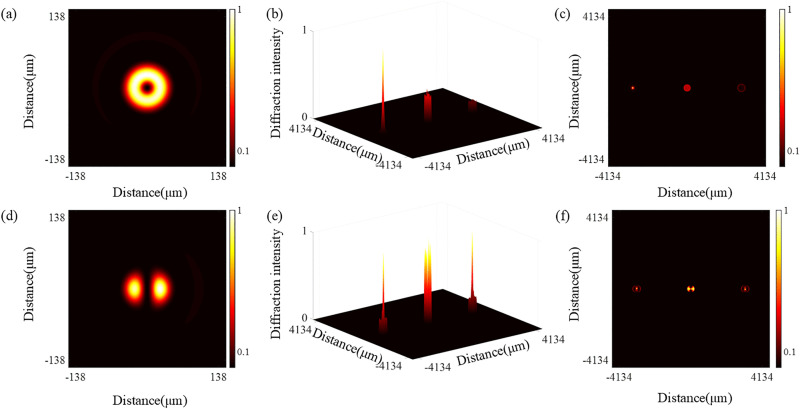
Simulation of optical communication multiplexing and demultiplexing. **(a, d)** Simulated carrier multiplexing patterns for the SZPs and QSGSZPs, respectively. The *x* and *y* axes represent spatial coordinates in the beam cross-section. **(b, c, e,** **f)** Demultiplexing patterns after a forked grating, shown in 3D (height represents intensity) and 2D (color represents intensity) views for the SZPs and QSGSZPs, respectively.

The helical phase of vortex beams endows photons with the orbital angular momentum, establishing their physical basis as high-dimensional data carriers and offering substantial advantages in multiplexed communication systems [38]. Therefore, numerical simulations were employed to investigate the applicability of the QSGSZPs in high-dimensional wavelength division multiplexing systems, focusing specifically on a quantitative evaluation of their channel capacity performance. As shown in Fig 8, carrier multiplexing can be achieved using a single diffractive optical element. For demultiplexing, a specially designed forked gratings is used to demodulate the signal by diffracting the incident vortex beam into distinct propagation directions. For the SZPs, when a vortex beam with a topological charge of *l* = 1 is diffracted by the fork gratings, a solid bright spot exhibiting plane-wave characteristics appears at the + 1 order diffraction, while all other orders form doughnut-shaped spots whose radii increase with the absolute value of the diffraction order. For the QSGSZPs, when vortex beams with topological charges of *l*_1_ = 1 and *l*_2_ = 0 are diffracted by the fork gratings, stable solid bright spots are formed at both the ± 1 orders diffraction. Therefore, the fork gratings can effectively separate OAM modes to achieve demultiplexing functionality. Additionally, the QSGSZPs incorporating dual orbital angular momentum modes significantly enhances the system channel capacity compared to the SZPs, laying a foundation for efficient and reliable transmission in high-dimensional optical communication systems.

## 4. Experiments

**Fig 9:**
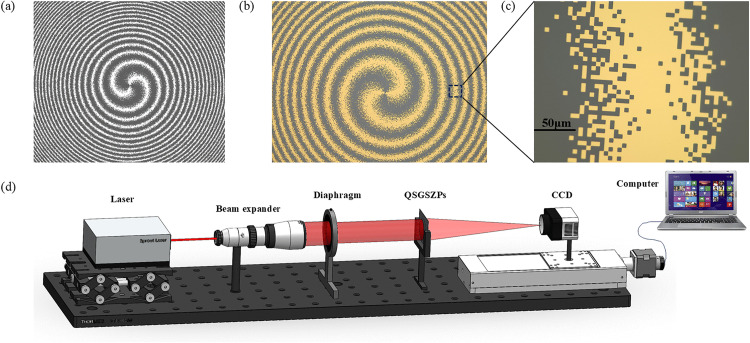
Fabrication process and experimental setup. **(a)** Design pattern of the QSGSZPs. **(b)** Experimental microscopy image of the fabricated sample on a quartz substrate. The scale bar indicates the spatial scale. **(c)** Enlarged experimental view of the area in the black rectangle in **(b)**, showing the fine binary square structure. **(d)** Schematic of the experimental setup. The optical path, from left to right, consists of a laser, a beam expander, a diaphragm, the QSGSZPs, a CCD camera, and a computer.

To validate the performance of the QSGSZPs, a proof-of-concept experiment was conducted. A series of the QSGSZPs with different topological charges were fabricated using the standard planar process, and the parameters for the QSGSZPs are set as: *λ* = 632.8 nm, the size *S* of square = 2.5 μm × 2.5 μm, *l*_1_ = 2, *l*_2_ = 2, and *f* = 200 mm. The fabrication procedure comprised the following key steps. Firstly, a 200-nm-thick chromium film was deposited on a quartz substrate via magnetron sputtering. Subsequently, a layer of photoresist AZ 5200 was spin-coated and soft-baked at 100°C for 120 seconds on a hotplate, resulting in a final resist thickness of approximately 1 μm. The photoresist was then exposed using a direct-write laser lithography system (DESIGN WRITE LAZER 2000) at a specific dose. After exposure, the sample was developed in a developer for approximately 40 seconds to achieve well-defined photoresist patterns. Using the photoresist as a mask, the chromium film was wet-etched at room temperature with a 10 wt% aqueous solution of ceric ammonium nitrate for about 90 seconds. Upon completion of etching, the sample was immersed in acetone and subjected to ultrasonic cleaning to remove residual photoresist, followed by rinsing with deionized water and drying with nitrogen gas. The image of the fabricated QSGSZPs is shown in Figs 9 (b)– 9(c), revealing that the binary structure is almost consistent with the designed pattern in Fig 9 (a). The excellent pattern transfer effect fully validates the good morphological control capability throughout the entire process.

The optical performance of the QSGSZPs was characterized using a self-built optical setup shown in Fig 9 (d). A helium-neon laser with the wavelength of 632.8 nm and the output power of 0.8 mW (Thorlabs HNLS008L) served as the coherent light source. Nextly, the output beams was first collimated and expanded by a 4 × beam expander, and spatially filtered by an adjustable diaphragm. The collimation and planarity of the wavefront were verified by examining the interference fringes using a shearing interferometer prior to the experiments. The collimated beam was normally incident onto the QSGSZPs, and the intensity distribution at the focal plane was finally recorded by a CCD camera (Lumenera LW230, 1600 × 1200 pixels, 4.4 μm pixel size), which was mounted on a high-precision translation stage to locate the focal plane precisely by moving along the optical axis.

**Fig 10:**
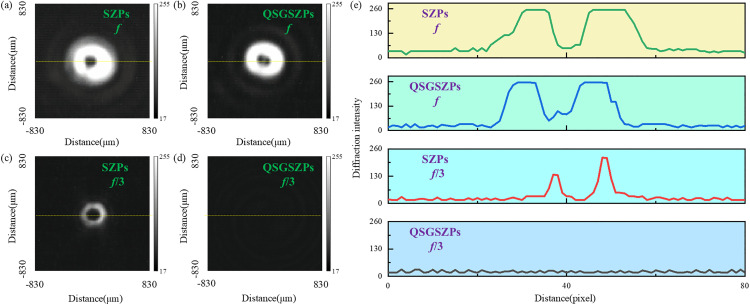
Experimental validation of high-order diffraction suppression. **(a) – (d)** The recorded intensity distributions of the SZPs and the QSGSZPs at the position of *f* and *f*/3, respectively. The x and y axes correspond to the pixel coordinates of the CCD, representing spatial position in the image plane. **(e)** The intensity distributions of the red dotted lines corresponding to **(a)** – **(d)**.

The diffraction intensity distributions of the SZPs and the QSGSZPs obtained by the CCD are shown in Fig 10. Due to the upper limit of the CCD grayscale, the first-order diffraction is actually oversaturated. As expected, in addition to generating optical vortex with a circular dark core structure at the position of *f*, high-order diffraction vortices are also clearly observed at the position of *f*/3 for the SZPs. In contrast, the desired first-order optical vortex for the QSGSZPs is equally clearly presented at the position of *f* shown in Fig 10 (c), while the high-order diffraction vortices are effectively suppressed in Fig 10 (d), leaving only background noise with an intensity almost identical to that of the SZPs. The experimental results demonstrate that the QSGSZPs outperforms the SZPs in suppressing high-order diffraction vortices and enhancing the purity of the target vortex, highlighting its promising potential for optical vortex generation and applications.

**Fig 11:**
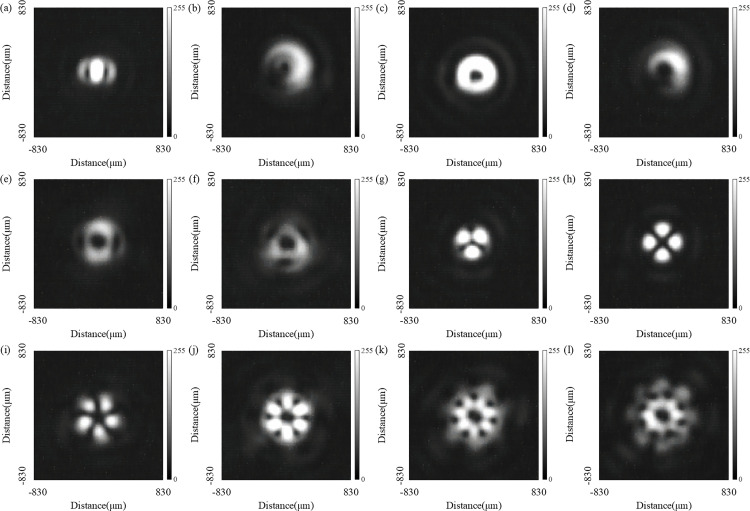
Experimental results of complex vortex generation. Recorded intensity distributions for the QSGSZPs with varying topological charges (*l*_1_, *l*_2_), corresponding to the simulations in Fig 5. **(a)** – **(l)** The topological charges of the QSGSZPs are as follows: (*l*_1_ = 2, *l*_2_ = 0), (*l*_1_ = 2, *l*_2_ = 1), (*l*_1_ = 2, *l*_2_ = 2), (*l*_1_ = 2, *l*_2_ = 3), (*l*_1_ = 2, *l*_2_ = 4), (*l*_1_ = 2, *l*_2_ = 5), (*l*_1_ = 2, *l*_2_ = −1), (*l*_1_ = 2, *l*_2_ = −2), (*l*_1_ = 2, *l*_2_ = −3), (*l*_1_ = 2, *l*_2_ = −4), (*l*_1_ = 2, *l*_2_ = −5), and (*l*_1_ = 2, *l*_2_ = −6), respectively. The parameters *l*_1_ is fixed at 2 while *l*_2_ is varied.

Similarly, the far-field diffraction patterns generated by the QSGSZPs with different topological charges are captured by the CCD, as shown in Fig 11. As the topological charge varies, it can be observed that the QSGSZPs can generate not only conventional optical vortices but also complex vortex structures such as the flower-shaped optical vortex lattice and vortex twins. The experimental results both visually demonstrate the capability of the QSGSZPs to modulate optical fields under different topological charges and provide experimental evidence for its potential applications in areas such as multi-mode optical communication and particle manipulation.

**Fig 12:**
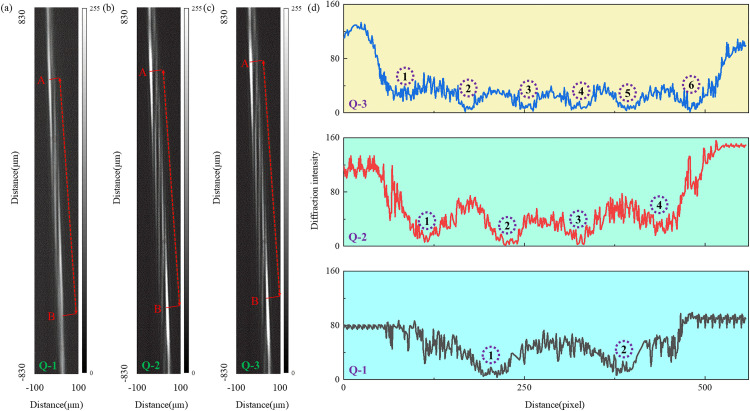
Experimental characterization of the topological charge. **(a) – (c)** Experimental interference patterns obtained after passing the vortex beams with topological charges of *l* = 2, = 4 and 6 through a cylindrical lens, respectively. (d) shows the intensity distribution along the red dashed line path between points A and B corresponding to **(a)** – **(c)**. The positions of the dark fringes correspond to the purple dashed circles, and the number of these circles corresponds to the topological charge.

To systematically verify the helical phase structure of the output beam, a cylindrical lens was placed between the QSGSZPs and the CCD. Vortex beams with topological charges of *l* = 2, 4, and 6 were successively passed through the cylindrical lens, where they interfered at the focal plane and formed distinct fringe patterns, which were ultimately captured and recorded by the CCD. As shown in Fig 12, the number of dark fringes in the obtained interference patterns directly corresponds to the integer value of the topological charge, while the overall tilt direction of the fringes reflects the positive or negative sign of the topological charge. Analysis of the fringe characteristics confirms that the topological charges of the three vortex beams correspond to 2, 4, and 6, respectively, showing complete consistency with the target design values. The experimental results unequivocally validate the effectiveness and reliability of the QSGSZPs in generating high-purity and controllable orbital angular momentum beams, further demonstrating its potential for structured light-field manipulation and related applications.

## 5. Conclusion

In summary, we have extended the concept of the the SZPs by proposing a novel diffractive optical element named QSGSZPs, which overcomes the inherent limitation in the SZPs. By transforming the three-dimensional structure of a Gabor spiral zone plates into a two-dimensional planar structure, the QSGSZPs achieves an approximately sinusoidal transmittance function, thereby significantly suppressing high-order diffraction. Furthermore, it enables the flexible generation of complex vortex structures, such as the flower-shaped optical vortex lattice and vortex twins through the adjustment of topological charge. Numerical simulations demonstrate that the focusing properties of the QSGSZPs can be optimized and controlled by appropriately selecting its parameters, and its potential applications in edge-enhanced imaging and optical communications were preliminarily demonstrated. The proposed QSGSZPs innovatively balances performance, functionality, and manufacturability, offering a versatile platform solution for applications requiring high-purity optical fields and complex wavefront manipulation.

## Supporting information

S1 Text The code for calculating diffraction efficiency in MTLAB. (DOCX)
