# Supplementary material for: Quasi-random square gabor spiral zone plates for high-order diffraction suppression and multi-functional optical vortex generation
Source: PLoS One. 2026 Feb 5;21(2):e0340251. doi: 10.1371/journal.pone.0340251 (PMC12875491; doi:10.1371/journal.pone.0340251)
Supplement: S1 Text — (DOCX) [file pone.0340251.s001.docx]

**Supporting Information**

The supporting information for this paper primarily consists of MATLAB code, and all simulation figures were generated from this code.

The code in Appendix 1 is used to generate the normalized far-field diffraction intensity distribution, phase distribution, and axial diffraction intensity distribution for the SZPs, GaborSZPs and QSGSZPs. By modifying the relevant parameters in the code, figures such as Figure 1, 2, 3, 4, 5 and 6, can be generated.

The code in Appendix 2 is used to validate the imaging performance for the SZPs, GaborSZPs and QSGSZPs. By modifying the relevant parameters in the code, figures such as Figure 7, can be generated.

The code in Appendix 3 is used to validate optical communication multiplexing and demultiplexing. By modifying the relevant parameters in the code, figures such as Figure 8, can be generated.

Appendix 1

clc

clear

lambda=0.6328

k0=2*pi/lambda;

focallength=200000;

Number=60;

floor(focallength*lambda/(4.0*deltar*deltar));

Radius=sqrt(Number*lambda*focallength);

Nx =2000;

Nc=floor(Nx/2)+1;

range1=2*Radius;

step1=range1/(Nx-1);

yp1=-range1/2:step1:range1/2;

xp1=-range1/2:step1:range1/2;

[x1,y1]=meshgrid(yp1,xp1);

position1 = x1 + 1i * y1;

lensletr = abs(position1);

lensang = angle(position1);

TC1st1=1;

TC1st2=1;

lens1=real(1/2-1/4*(exp(-1i*pi*lensletr.*lensletr/(lambda*focallength)+TC1st1*1i*lensang)+exp(-1i*pi*lensletr.*lensletr/(lambda*focallength)+TC1st2*1i*lensang))).*(1-sign(lensletr-Radius))/2;

figure(1)

imshow(lens1)

lens2=zeros(Nx,Nx);

for i=1:Nx/2

for j=1:Nx/2

pinjun=(lens1(2*(i-1)+1,2*(j-1)+1)+lens1(2*(i-1)+1,2*(j-1)+2)+lens1(2*(i-1)+2,2*(j-1)+1)+lens1(2*(i-1)+2,2*(j-1)+2))/4;

if pinjun>=0 && 0.05>pinjun

num1=0.1;

else if pinjun>=0.05 && 0.1>pinjun

num1=0.1;

else if pinjun>=0.1 && 0.15>pinjun

num1=0.1;

else if pinjun>=0.15 && 0.2>pinjun

num1=0.1;

else if pinjun>=0.2 && 0.25>pinjun

num1=0.3;

else if pinjun>=0.25 && 0.3>pinjun

num1=0.3;

else if pinjun>=0.3 && 0.35>pinjun

num1=0.3;

else if pinjun>=0.35 && 0.4>pinjun

num1=0.3;

else if pinjun>=0.4 && 0.45>pinjun

num1=0.5;

else if pinjun>=0.45 && 0.5>pinjun

num1=0.5;

else if pinjun>=0.5 && 0.55>pinjun

num1=0.5;

else if pinjun>=0.55 && 0.6>pinjun

num1=0.5;

else if pinjun>=0.6 && 0.65>pinjun

num1=0.7;

else if pinjun>=0.65 && 0.7>pinjun

num1=0.7;

else if pinjun>=0.7 && 0.75>pinjun

num1=0.7;

else if pinjun>=0.75 && 0.8>pinjun

num1=0.7;

else if pinjun>=0.8 && 0.85>pinjun

num1=0.9;

else if pinjun>=0.85 && 0.9>pinjun

num1=0.9;

else if pinjun>=0.9 && 0.95>pinjun

num1=0.9;

else

num1=0.9;

end

end

end

end

end

end

end

end

end

end

end

end

end

end

end

end

end

end

end

num2=rand;

if pinjun >= num2

lens2(2*(i-1)+1,2*(j-1)+1)=1;

lens2(2*(i-1)+1,2*(j-1)+2)=1;

lens2(2*(i-1)+2,2*(j-1)+1)=1;

lens2(2*(i-1)+2,2*(j-1)+2)=1;

else

lens2(2*(i-1)+1,2*(j-1)+1)=0;

lens2(2*(i-1)+1,2*(j-1)+2)=0;

lens2(2*(i-1)+2,2*(j-1)+1)=0;

lens2(2*(i-1)+2,2*(j-1)+2)=0;

end

end

end

lens12=sign(1+sign(range1/2*range1/2-x1.*x1-y1.*y1));

lens2=lens2.*lens12;

figure(2)

imshow(lens2)

SZP=exp(-1i*k0*lensletr.^2/(2*focallength)+0.5*1i*pi+TC1st1*1i*lensang).*(1-sign(lensletr-Radius))/2;

lens3 =sign(1+sign(real(SZP)));

lens3=1-lens3;

figure(3)

imshow(lens3)

% area1=range1*0.05;

% object1=lens2;

% distance1=focallength;

% E_out1=propagor_czt(object1,Nx,lambda,range1,distance1,area1);

I_out1=abs(E_out1).^2;

figure(7)

mesh(x1,y1,I_out1);

figure(8)

imagesc(xp1,yp1,I_out1);colorbar;

axis off

az=angle(E_out1);

figure(9)

imagesc(yp1,xp1,az);colorbar;

www1=linspace(20000,250000,Nx);

NUM=0;

for i=1:Nx

area1=range1*0.05;

object1=lens2;

distance1=www1(i);

E_out1=propagor_czt(object1,Nx,lambda,range1,distance1,area1);

I_out1=abs(E_out1).^2;

NUM=NUM+1

figure(10)

imagesc(xp1,yp1,I_out1);colorbar;

axis off

c1=I_out1(Nc,1:Nx);

zzz(:,i)=c1;

end

figure(11)

zzz=zzz/max(max(zzz));

imagesc(xp1,yp1,zzz);colorbar;

axis off

[maxValue, idx] = max(zzz(:));

[row, col] = ind2sub(size(zzz), idx);

figure(12)

c2=zzz(row,:);

plot(www1,c2)

Appendix 2

TC1st=2;

lambda=0.6;

k0=2*pi/lambda;

focallength1=300000;

deltar=3;

Number=200;

Radius=sqrt(200*lambda*focallength1);

Nx =4000;

Nc=floor(Nx/2)+1;

range1=2*Radius;

step1=range1/(Nx-1);

yp1=-range1/2:step1:range1/2;

xp1=-range1/2:step1:range1/2;

[y1,x1]=meshgrid(yp1,xp1);

position1 = x1 + 1i * y1;

lensletr = abs(position1);

lensang = angle(position1); 

%% Rectangle

% R1=40;

% Y1=sign(1+sign(y1+R1));

% Y2=sign(1+sign(R1-y1));

% Y3=sign(1+sign(x1+R1));

% Y4=sign(1+sign(R1-x1));

% Y=Y1.*Y2.*Y3.*Y4;

% figure(1)

% imshow(Y)

%% circle

R1=50;

Y1=sign(1+sign(pi*R1*R1-x1.*x1-y1.*y1));

Y=Y1;

figure(1)

imshow(Y)

%% Semicircle

% R1=60;

% Y=sign(1+sign(pi*R1*R1-x1.*x1-y1.*y1)).*sign(1+sign(y1));

% figure(1)

% imshow(Y)

%% Triangle

% R1=60;

% Y1=sign(1+sign(-y1+x1+R1));

% Y2=sign(1+sign(y1+x1+R1));

% Y=Y1.*Y2.*sign(1+sign(-x1+R1/2));

% figure(1)

% imshow(Y)

z1=4*pi*R1*R1/lambda;

area1=range1;

object1=Y;

distance1=focallength1;

E_out1=propagor_czt(object1,Nx,lambda,range1,distance1,area1);

I_out1=abs(E_out1).^2;

figure(2)

mesh(x1,y1,I_out1);

lens1 = exp(-1i*k0*lensletr.^2/(2*focallength1)).*(1-sign(lensletr-Radius))/2;

figure(3)

imshow(lens1)

E_after_lens1 = E_out1.*lens1;

distance2=2*focallength1;

E_fourier=propagor_czt(E_after_lens1,Nx,lambda,range1,distance2,area1);

focallength=200000;

TC1st1=2;

TC1st2=2;

lens3=real(1/2-1/4*(exp(-1i*pi*lensletr.*lensletr/(lambda*focallength)+TC1st1*1i*lensang)+exp(-1i*pi*lensletr.*lensletr/(lambda*focallength)+TC1st2*1i*lensang))).*(1-sign(lensletr-Radius))/2;

figure(4)

imshow(lens3)

TFZP2=exp(-1i*k0*lensletr.^2/(2*focallength)+0.5*1i*pi+2*1i*lensang).*(1-sign(lensletr-Radius))/2;

lens6 =sign(1-sign(real(TFZP2)));

figure(5)

imshow(lens6)

TFZP7= exp(-1i*k0*lensletr.^2/(2*focallength)).*(1-sign(lensletr-Radius))/2;

lens7 =sign(1-sign(real(TFZP7)));

figure(6)

imshow(lens7)

lens2=zeros(Nx,Nx);

for i=1:Nx/2

for j=1:Nx/2

pinjun=(lens3(2*(i-1)+1,2*(j-1)+1)+lens3(2*(i-1)+1,2*(j-1)+2)+lens3(2*(i-1)+2,2*(j-1)+1)+lens3(2*(i-1)+2,2*(j-1)+2))/4;

if pinjun>=0 && 0.05>pinjun

num1=0.1;

else if pinjun>=0.05 && 0.1>pinjun

num1=0.1;

else if pinjun>=0.1 && 0.15>pinjun

num1=0.1;

else if pinjun>=0.15 && 0.2>pinjun

num1=0.1;

else if pinjun>=0.2 && 0.25>pinjun

num1=0.3;

else if pinjun>=0.25 && 0.3>pinjun

num1=0.3;

else if pinjun>=0.3 && 0.35>pinjun

num1=0.3;

else if pinjun>=0.35 && 0.4>pinjun

num1=0.3;

else if pinjun>=0.4 && 0.45>pinjun

num1=0.5;

else if pinjun>=0.45 && 0.5>pinjun

num1=0.5;

else if pinjun>=0.5 && 0.55>pinjun

num1=0.5;

else if pinjun>=0.55 && 0.6>pinjun

num1=0.5;

else if pinjun>=0.6 && 0.65>pinjun

num1=0.7;

else if pinjun>=0.65 && 0.7>pinjun

num1=0.7;

else if pinjun>=0.7 && 0.75>pinjun

num1=0.7;

else if pinjun>=0.75 && 0.8>pinjun

num1=0.7;

else if pinjun>=0.8 && 0.85>pinjun

num1=0.9;

else if pinjun>=0.85 && 0.9>pinjun

num1=0.9;

else if pinjun>=0.9 && 0.95>pinjun

num1=0.9;

else

num1=0.9;

end

end

end

end

end

end

end

end

end

end

end

end

end

end

end

end

end

end

end

num2=rand;

if pinjun >= num2

lens2(2*(i-1)+1,2*(j-1)+1)=1;

lens2(2*(i-1)+1,2*(j-1)+2)=1;

lens2(2*(i-1)+2,2*(j-1)+1)=1;

lens2(2*(i-1)+2,2*(j-1)+2)=1;

else

lens2(2*(i-1)+1,2*(j-1)+1)=0;

lens2(2*(i-1)+1,2*(j-1)+2)=0;

lens2(2*(i-1)+2,2*(j-1)+1)=0;

lens2(2*(i-1)+2,2*(j-1)+2)=0;

end

end

end

lens12=sign(1+sign(range1/2*range1/2-x1.*x1-y1.*y1));

lens2=lens2.*lens12;

figure(7)

imshow(lens2)

z2=pi*Radius*Radius/lambda;

area1=range1*0.018;

object1=lens2.*E_fourier;

distance1=focallength/3;

E_out2=propagor_czt(object1,Nx,lambda,range1,distance1,area1);

I_out2=abs(E_out2).^2;

I_out2=I_out2/max(max(I_out2));

figure(8)

mesh(x1,y1,I_out2);

figure(9)

imagesc(xp1,yp1,I_out2);colorbar;

axis off

Appendix 3

lambda=0.6328;

k0=2*pi/lambda;

focallength=200000;

Number=60; floor(focallength*lambda/(4.0*deltar*deltar));

Radius=sqrt(Number*lambda*focallength);

Nx =2000;

Nc=floor(Nx/2)+1;

range1=2*Radius;

step1=range1/(Nx-1);

yp1=-range1/2:step1:range1/2;

xp1=-range1/2:step1:range1/2;

[x1,y1]=meshgrid(yp1,xp1);

position1 = x1 + 1i * y1;

lensletr = abs(position1);

lensang = angle(position1); 

TC1st1=1;

TC1st2=-1;

lens1=real(1/2-1/4*(exp(-1i*pi*lensletr.*lensletr/(lambda*focallength)+TC1st1*1i*lensang)+exp(-1i*pi*lensletr.*lensletr/(lambda*focallength)+TC1st2*1i*lensang))).*(1-sign(lensletr-Radius))/2;

figure(1)

imshow(lens1)

lens2=zeros(Nx,Nx);

for i=1:Nx/2

for j=1:Nx/2

pinjun=(lens3(2*(i-1)+1,2*(j-1)+1)+lens3(2*(i-1)+1,2*(j-1)+2)+lens3(2*(i-1)+2,2*(j-1)+1)+lens3(2*(i-1)+2,2*(j-1)+2))/4;

if pinjun>=0 && 0.05>pinjun

num1=0.1;

else if pinjun>=0.05 && 0.1>pinjun

num1=0.1;

else if pinjun>=0.1 && 0.15>pinjun

num1=0.1;

else if pinjun>=0.15 && 0.2>pinjun

num1=0.1;

else if pinjun>=0.2 && 0.25>pinjun

num1=0.3;

else if pinjun>=0.25 && 0.3>pinjun

num1=0.3;

else if pinjun>=0.3 && 0.35>pinjun

num1=0.3;

else if pinjun>=0.35 && 0.4>pinjun

num1=0.3;

else if pinjun>=0.4 && 0.45>pinjun

num1=0.5;

else if pinjun>=0.45 && 0.5>pinjun

num1=0.5;

else if pinjun>=0.5 && 0.55>pinjun

num1=0.5;

else if pinjun>=0.55 && 0.6>pinjun

num1=0.5;

else if pinjun>=0.6 && 0.65>pinjun

num1=0.7;

else if pinjun>=0.65 && 0.7>pinjun

num1=0.7;

else if pinjun>=0.7 && 0.75>pinjun

num1=0.7;

else if pinjun>=0.75 && 0.8>pinjun

num1=0.7;

else if pinjun>=0.8 && 0.85>pinjun

num1=0.9;

else if pinjun>=0.85 && 0.9>pinjun

num1=0.9;

else if pinjun>=0.9 && 0.95>pinjun

num1=0.9;

else

num1=0.9;

end

end

end

end

end

end

end

end

end

end

end

end

end

end

end

end

end

end

end

num2=rand;

if pinjun >= num2

lens2(2*(i-1)+1,2*(j-1)+1)=1;

lens2(2*(i-1)+1,2*(j-1)+2)=1;

lens2(2*(i-1)+2,2*(j-1)+1)=1;

lens2(2*(i-1)+2,2*(j-1)+2)=1;

else

lens2(2*(i-1)+1,2*(j-1)+1)=0;

lens2(2*(i-1)+1,2*(j-1)+2)=0;

lens2(2*(i-1)+2,2*(j-1)+1)=0;

lens2(2*(i-1)+2,2*(j-1)+2)=0;

end

end

end

lens12=sign(1+sign(range1/2*range1/2-x1.*x1-y1.*y1));

lens2=lens2.*lens12;

figure(2)

SZP=exp(-1i*k0*lensletr.^2/(2*focallength)+0.5*1i*pi+TC1st1*1i*lensang).*(1-sign(lensletr-Radius))/2;

lens3 =sign(1+sign(real(SZP)));

lens3=1-lens3;

figure(4)

imshow(lens3)

area1=range1*0.04;

object1=lens1;

distance1=focallength;

E_out1=propagor_czt(object1,Nx,lambda,range1,distance1,area1);

I_out1=abs(E_out1).^2;

figure(7)

mesh(x1,y1,I_out1);

I_out1=I_out1/max(max(I_out1));

figure(8)

imagesc(xp1,yp1,I_out1);colorbar;

axis off

az=angle(E_out1);

figure(9)

imagesc(yp1,xp1,az);colorbar;

d1=80;

TC3st=1;

phi3=exp(-1i*(2*pi*x1./(d1)+TC3st*lensang));

lensfk=sign(1+sign(real(phi3)));

area1=range1*2;

object1=lensfk.*E_out1;

distance1=150000;

E_out2=propagor_czt(object1,Nx,lambda,range1,distance1,area1);

I_out2=abs(E_out2).^2;

figure(11)

mesh(x1,y1,I_out2);

figure(12)

imagesc(xp1,yp1,I_out2);colorbar;

axis off

focallength1=180000;

lens6 = exp(-1i*k0*lensletr.^2/(2*focallength1)).*(1-sign(lensletr-Radius))/2;

figure(13)

imshow(lens6)

area1=range1*1.5;

object1=lens6.*E_out2;

distance1=focallength1;

E_out3=propagor_czt(object1,Nx,lambda,range1,distance1,area1);

I_out3=abs(E_out3).^2;

I_out3=I_out3/max(max(I_out3));

I_out1=I_out1/max(max(I_out1));

yp2=-range1*1.5/2:step1:range1*1.5/2;

xp2=-range1*1.5/2:step1:range1*1.5/2;

[x2,y2]=meshgrid(yp2,xp2);

figure(14)

mesh(x1,y1,I_out3);

axis on

figure(16)

imagesc(xp1,yp1,I_out3);colorbar;

axis off

Appendix 4(propagor_czt.m)

function [gout] = propagor_czt(object,Nx,lambda,range,distance,area)

din=range/(Nx-1);

dout=area/(Nx-1);

k0=2*pi/lambda;

[y,x] = meshgrid(-range/2:din:range/2,-range/2:din:range/2);

rzp=sqrt(x.^2+y.^2);

fs=lambda*distance/din;

fy1=-area/2+fs/2;

fy2=area/2+fs/2;

fx1=-area/2+fs/2;

fx2=area/2+fs/2;

object=object.*exp(1i*k0/(2*distance)*rzp.^2);

a = -exp(1j*2*pi*fy1/fs);

w = exp(-1j*2*pi*(fy2-fy1)/(Nx*fs));

gout = czt(object,Nx,w,a);

lly=linspace(0,Nx-1,Nx);lly=lly./Nx.*(fy2-fy1)+fy1;

Mshifty=floor(-Nx/2);

Mshift=repmat(exp(-1i.*2*pi.*lly.*Mshifty/fs),[Nx 1]);

gout=gout.'.*Mshift;

a = -exp(1j*2*pi*fx1/fs);

w = exp(-1j*2*pi*(fx2-fx1)/(Nx*fs));

gout = czt(gout,Nx,w,a);

llx=linspace(0,Nx-1,Nx);llx=llx./Nx.*(fx2-fx1)+fx1;

Mshiftx=floor(-Nx/2);

Mshift=repmat(exp(-1i.*2*pi.*llx.*Mshiftx/fs),[Nx 1]);

gout=gout.'.*Mshift;

[ys,xs] = meshgrid(-area/2:dout:area/2,-area/2:dout:area/2);

rs2 = xs.^2+ys.^2;

F0=exp(1j*k0*distance)/(1j*lambda*distance).*exp(1j*k0/2/distance*rs2);

gout=F0.*gout*din^2;

end

Appendix 5(czt.m)

function g=czt(x,k,w,a)

[m,n]=size(x);

nfft=2^nextpow2(m+k-1);

kk=((-m+1):max(k-1,m-1)).';

ww=w.^((kk.^2)./2);

nn=(0:(m-1))';

aa=a.^(-nn).*ww(m+nn);

y=x.*aa(:,ones(1,n));

fy=fft(y,nfft);

fv=fft(1./ww(1:(k-1+m)),nfft);

fy=fy.*fv(:,ones(1,n));

g=ifft(fy);

g=g(m:(m+k-1),:).*ww(m:(m+k-1),ones(1,n));

end
